# Survey of the situation of the prehospital emergency medical services system in Iran

**DOI:** 10.1186/s12873-025-01350-5

**Published:** 2025-10-29

**Authors:** Mohammad Esmail Tavakoli Abandansari, Saeed Mehrsourosh, Mehdi Jafari Sirizi, Hamidreza Khankeh, Khatereh Mohammadi, Khosro Shakeri

**Affiliations:** 1 Tehran Province Emergency Organization, Tehran, Iran; 2 Faculty of Medical Management and Informatics, Iran University of Medical Sciences, Tehran, Iran; 3 School of Social Health, Center for Health Research in Accidents and Disasters, University of Rehabilitation Sciences and Social Health, Tehran, Iran; 4 School of Medical Management and Informatics, Iran University of Medical Sciences, Tehran, Iran

**Keywords:** Prehospital emergency medical services, Health service delivery, Qualitative research, Content analysis, EMS system evaluation

## Abstract

**Objective:**

The present study investigated the status of prehospital emergency medical services in Iran in 1402.

**Research method:**

To examine the current structure and components of Iran’s prehospital emergency medical services system, we conducted a qualitative study using content analysis through semi-structured interviews with 38 experts, managers, and service providers across the country. The interview guide was developed based on a review of related literature and consultations with specialists in the field. Data were analyzed using a conventional content analysis approach, and the analysis was carried out with the help of MAXQDA10 software.

**Findings:**

The findings of the present study are presented in the form of 8 themes (Rules and Policies and policies, human resource management, infrastructure, challenges of public education and information, service delivery process, responsible organization, and service delivery model).

**Conclusion:**

The current service delivery model does not meet the needs of society and needs to be changed in the existing model. Therefore, it is suggested that by strengthening the Independence House at the level of the ministry and universities, providing services with a hybrid model, piloting prehospital emergency medical services by other institutions from families, municipalities, special departments in regions of the country and strengths and weaknesses and comparing it with the current situation and reflecting its results at the level of national services, prehospital services.

**Supplementary Information:**

The online version contains supplementary material available at 10.1186/s12873-025-01350-5.

## Introduction

Several organizations have been established to protect people’s health, each performing a part of this task. One of these important organizations that plays a significant and fundamental role in responding to emergencies and accidents and preventing deaths and disabilities is the prehospital emergency [1]. The emergency medical services (EMS) system is one of the vital services of society. This system provides emergency medical services from the patient’s bedside to the hospital emergency room. The medical services provided include trauma patients who have been injured as a result of an accident and inpatients who have no history of trauma [2]. The four primary functions of this system in the prehospital stage are performing triage and providing essential care to preserve the lives of patients, preventing further injuries to patients and injured people, rapid transfer to an appropriate medical center and care during transfer, and providing appropriate reports on the type of accident and the condition of patients transferred to the medical center [3]. Studies show that traffic accidents are the leading cause of illness/injury in both sexes in Iran. Studies show that rapid response and appropriate, quality treatment in this system can reduce deaths from accidents by up to 30% and prevent complications resulting from inappropriate actions by non-professionals [4]. Today, in the urban health system, the initial examination and treatment of critical patients is generally carried out by the prehospital emergency department. The more correct, accurate, and rapid this approach is, the lower the mortality and disability rates from diseases will be, and the more people will trust this system [5]. In recent years, approaches to the prehospital emergency system have changed, such that the World Health Organization (WHO) considers the prehospital emergency system an integral part of any effective and efficient health care system [6]. By providing appropriate prehospital services, the lives of more than 1.2 million people can be saved annually, and more than 50 million disabilities can be prevented [7].

Trauma is also the most common cause of death among the age group under 50 years. Road accidents alone are responsible for more than 24,000 deaths per year in this country [8]. According to the World Health Organization report, it has been proven that a systemic approach is needed to reduce casualties and disability caused by injuries. This systemic approach, known as the trauma system, covers a wide range of care, including prehospital, hospital, and post-hospital care [9]. To the extent that prehospital emergency services and their improvement are mentioned as one of the main factors in reducing the burden caused by accidents [9, 10]. In order to strengthen the prehospital system in responding effectively and timely to accidents and disasters and those in need of emergency medical services, and to prevent short-term and permanent disabilities resulting from injuries and trauma, and to reduce the burden of accidents and other medical problems, and to increase the satisfaction of service providers in this area, it is essential to identify the strengths and areas for improvement of the existing system. Therefore, this study aims to examine the status of the prehospital emergency medical services system in Iran by extracting national experiences in the field of prehospital emergency.

## Methods

The present study is qualitative research conducted to identify the current status and dimensions of Iran’s prehospital emergency medical services system in 2023. The research community consisted of experts in the field of prehospital emergency (see Supplementary Material 1), faculty members affiliated with emergency departments at medical universities, managers and operational staff working in prehospital and hospital emergency departments across the country, staff of the Ministry of Health and the National Emergency Organization, and service recipients.

The sampling method was purposive, and the method of “maximum diversity sampling” and participants were selected from different levels of organizational positions, including technicians and experts directly providing the service, middle managers, senior managers, policymakers, and faculty members from different cities and provinces across the country, who had experience in this regard, as well as service recipients. The reason for interviewing service recipients was the need for information identified during the study. Interviews continued until data saturation was reached. The inclusion criteria for participants in the study were having experience in prehospital emergency and interest in participation, and the exclusion criterion included unwillingness to continue cooperation.

To conduct the interviews, a semi-structured interview guide was designed based on a review of relevant studies and the opinions of specialists and experts in this field. The interview guide was developed in four types: interview guides for executive managers, senior managers, policymakers and scholars, operational staff, and prehospital emergency service users. The content validity of the interview guide was confirmed by three experts in qualitative research and prehospital emergency medical services prior to data collection. (The semi-structured interview guide used for data collection was developed specifically for this study and is provided as a supplementary file 2. It includes four representative questions for each participant group: executive managers, policymakers and scholars, operational staff, and service users). According to the research analysis framework and study objectives, the questions were asked of executive managers about their experiences and management problems in this area and providing solutions, of senior managers, policymakers, and scholars about their experiences and significant problems in this area and providing solutions based on their studies and scientific knowledge. Operational staff were asked questions that were appropriate to their understanding of the experience and problems they faced in providing services and the proposed solutions. Finally, the public and service users were asked about their experiences receiving services, problems, and expectations from an EMS system. Auxiliary questions were used to clarify and guide the research topic if necessary. In addition to the interviews, information from a two-day meeting of the managers of the medical emergency and disaster management centers across the country at the National Emergency Organization and messages from technicians from across the country on social media were collected, and codes were extracted that contributed significantly to the richness of the findings.

Prior to conducting all interviews with the participants, prior coordination was made. After attending the designated time and place and providing the necessary explanations regarding the research objectives and the importance and necessity of conducting it, the interviews were conducted after obtaining informed and voluntary consent from the participants. Thus, in the presence of the interviewee, both the Sony recorder and the mobile phone recording mode were turned on simultaneously. When turned on and recorded, it was explained to them that their interview would be recorded for implementation. During the interview, feedback techniques and probing questions were used to clarify and resolve ambiguities in some cases. The interviewees’ responses were listened to without any prejudice or judgment, and new questions were asked without direction in line with the research objectives. Important points were noted on a notepad to prevent loss of information due to the recorder’s failure. All interviews were conducted by trained specialists with relevant academic backgrounds in emergency medical services and health management. Data analysis was led by a professor with a PhD and expertise in qualitative methodology, supported by a master’s student who assisted in transcription and coding. This collaboration ensured a rigorous and systematic approach to qualitative data collection and analysis.

The method used in this study for data analysis was the content analysis method. Given that the existing knowledge about the status and pattern of prehospital emergency services in the country and the health system was very small, vague, and scattered, and also that opinions and literature reviews on the phenomenon in question in the country are limited, Data analysis was conducted using a conventional content analysis approach. The process followed three main stages: preparation, organization, and reporting [11]. After implementation, the writings were read several times, and, as far as possible, meaning units and codes were extracted to enable the description of all aspects of the content. Then, a summary or compression process was performed, and the codes that were very close in meaning were placed into subcategories, and a list of subcategories was prepared. These subcategories were then grouped into larger headings, and finally, the results were reported in the form of themes, main categories, subcategories, codes, and related meaning units. MAXQDA10 software was used to perform the data analysis process.

In qualitative research, the concepts of acceptability, reliability, verifiability, and transferability are used to express the accuracy and robustness of the data [12]. In this study, various methods were used to confirm the acceptability of the data, including rethinking the purpose of the study, careful selection of key informants, especially from among the managers of prehospital emergency centers, using an in-depth interview method with open-ended questions to collect information, rethinking the semantic units for analysis, engaging with the data for a long time and spending sufficient time to collect and analyze the data, fully explaining how to create the units of meaning, compressing and abstracting the main categories, using quotes in the report of findings for documentation, analysis, and interpretations to explain the hidden meanings, reviewing the categories by the participants and the research team, reviewing the data and their analysis process by the research team, using peer review, using interviews, observation, and note-taking in data collection. To increase the reliability of the data, interviews were conducted over a specific and continuous period, with maximum effort to maintain conditions related to focusing on the topic and similar questions appropriate to the interviewees’ positions. Data analysis and code classification were also carefully reviewed by an external reviewer familiar with and experienced in the field of conventional content analysis. Continuous comparisons between the content of the categories and subcategories were used to monitor semantic and structural coherence. Also, analogy and induction were carried out in each category to ensure semantic and structural coherence. In order to achieve verifiability, all stages of the research, especially the stages of data analysis, were recorded in detail throughout the entire process so that if another researcher wishes to continue research in this area, he or she can easily follow this work based on the existing documents and documents related to the interviews and the way the categories are classified and named. To facilitate the transferability of the findings, a clear description of the context, selection method, participant characteristics, data collection process, and data analysis process was provided.

## Results

Thirty-eight managers and employees of the country’s medical emergency and disaster management centers, the National Emergency Organization, and five employees were interviewed individually. The interviewees were divided into four groups: (1) executive managers, (2) senior managers, policymakers, scientists, (3) technicians, and (4) employees. Specific questions were asked according to each group. It should be noted that each of these officials had at least one year of work experience in the mentioned organizational position. The results were extracted and analyzed through interviews, field observations, participation in managers’ meetings, and related notes from technicians on social networks. (The characteristics of the interviewees are reported in Table 1).

**Table 1:** Characteristics of the interviewees

NO.	Participant	Organizational Position	Education and Field	Work Experience in this Position (Years)	Work Experience (Years)	Interview Duration (Minutes)
**Heads and managers of pre-hospital emergency centers**						
1	1	Head of the County Emergency and Medical Emergency Management Center	Doctoral student in health in emergencies and disasters	1	20	40
2	12	Head of the University of Medical Sciences Emergency and Medical Emergency Management Center	Master of Nursing	7	21	55
3	13	Head of the University of Medical Sciences Emergency and Medical Emergency Management Center	Emergency Medicine Specialist	6 months	3	50
4	11	Head of the University of Medical Sciences Emergency and Medical Emergency Management Center	General Practitioner and Doctoral student in health in emergencies and disasters	1	7	90
5	16	Head of the University of Medical Sciences Emergency and Medical Emergency Management Center	General Practitioner and Doctoral student in health in emergencies and disasters	1	15	30
6	24	Head of the University of Medical Sciences Emergency and Medical Emergency Management Center	Emergency Medicine Specialist	1. 5	5	30
7	26	Head of the University of Medical Sciences Emergency and Medical	General Practitioner and Doctoral student in health in emergencies and disasters	1	12	38
8	26	Head of the County Emergency Medical Management Center	General Practitioner	2	6	78
9	18	Deputy Director of Operations, County Emergency Medical Management Center	Doctoral student in health in emergencies and disasters	1	11	78
10	35	County Emergency Manager	Master of Management	6	20	50
11	38	County Network Manager	General Practitioner and Doctoral student in health policy	8 months	20	20
**Senior managers and policymakers**						
12	34	Head of the National Emergency Organization	Doctor of Management	3	25	60
13	28	Faculty of the University of Medical Sciences (former head of the National Emergency Organization)	Specialist in Emergency Medicine	2	18	72
9	31	Faculty of the University of Medical Sciences (former head of the National Emergency Organization)	Specialist in Emergency Medicine	3	20	76
15	36	Advisor to the Head of the National Emergency Organization	Postdoctoral Fellowship in health in emergencies and disasters	4	27	40
16	15	Head of Department in the National Emergency Organization	General Practitioner	10	20	46
17	37	Head of Department in the National Emergency Organization	General Practitioner	5	20	20
18	21	Deputy Head of the National Emergency Organization	Specialist in Emergency Medicine	2	17	28
19	17	Head of Department in the National Emergency Organization	General Practitioner	4	21	40
20	32	Director of the Iranian Red Crescent Society	General Practitioner and Doctor of health in emergencies and disasters	3 ماه	25	85
21	23	Head of Department in the National Emergency Organization	Nursing Expert	3	12	50
22	27	Deputy Head of Education and Faculty of Nursing and Midwifery, University of Medical Sciences	Doctor of health in emergencies and disasters	8	20	50
23	33	Faculty of the Faculty of Emergency Medicine	Doctor of Nursing Student	4	6	20
24	19	Faculty and Director of the Emergency Department of the Faculty of Medical Sciences	Doctor of health in emergencies and disasters Student	4	7	20
25	29	Responsible for Emergency Research in a City	Nursing Expert	1	17	25
**Technicians**						
26	5	Emergency Medical Technician and Firefighter	Disaster and Emergency Specialist	12		73
27	2	Emergency Medical Technician	Master of Management	16		73
28	8	Emergency Medical Technician	Anesthesiologist	16		40
29	6	Emergency Medical Technician	Emergency Medical Specialist	6		65
30	7	Emergency Medical Technician	Emergency Medical Specialist	7		70
31	22	Emergency Medical Technician	Nurse	12		30
32	30	Emergency Medical Paramedic	PhD Student in health in emergencies and disasters	15		70
33	10	Emergency Medical Technician	Diploma	6		30
34	9	Emergency Medical Technician	Emergency Medical Specialist	10		60
35	20	Emergency Medical Technician	PhD Student in Health Economics	10		87
36	14	Emergency Medical Technician	Emergency Specialist	9		42
37	4	Emergency Medical Technician	Emergency Specialist	9		63
38	3	Emergency Medical Technician and Firefighter	Disaster and Emergency Specialist	28		63
**Servants**						
39	39	Employee	Master			20
40	40	Soldier	Cycle			15
41	41	Housewife	Diploma			15
42	42	Freelance	Master			10
43	43	Freelance	Diploma			18

A total of 43 individuals participated in the study, including executive managers, policymakers, technicians, and service users. Their educational backgrounds ranged from diploma holders to PhD students, and their professional experience varied from 1 to 28 years.

In the first stage of data analysis, 1300 open codes were extracted. In the subsequent stages, and by continuing the process of continuous comparison, classification based on common points and elimination of repetitive codes and concepts, 800 codes, 65 subcategories, 27 categories, and 8 themes or themes (laws and policies, human resource management, infrastructure, challenges of public education and information, service delivery process, interaction, responsible organization, and service delivery model) was formed. The codes and concepts are displayed in more detail in Appendix 1.

### First theme: Laws and policies

The first theme resulting from the research is laws and policies, which relate to the set of laws, general policies, and pre-hospital emergency protocols and help guide the emergency organization. This theme includes two layers of policy (three sub-layers of treatment prioritizing prevention, centralization, and a specialized education system) and laws and protocols that restrict service (four sub-layers of opaque administrative and financial laws, professional and trade laws, lack of SOPs, and laws that restrict service).

“One of the strengths of the emergency department is that the emergency department structure is the same in many parts of the country.” (Interviewee 29).

“I disagree with the system moving away from an integrated state. The more integrated it becomes, the better for the system because our country does not have an ideal distribution of economic resources. It should not move away from a state of income concentration.” (Interviewee 31).

Another participant mentioned that laws and regulations exist but need to be reviewed.

“This criterion of one base for every 40,000 people (2 ambulances for urban bases, one ambulance for roadside bases) should be changed. The criterion should be access to ambulances. People should have access to ambulance services within 4–5 minutes. The number of bases should be proportional to the number of incidents and needs, not just based on population.” (Interviewee 11).

### Second theme: Human resource management

The second theme from the research is related to issues and problems related to human resources. Human resources are the most important competitive advantage and organizational capital. Human resources are the most important element of any organization, especially in pre-hospital emergency care. For this reason, it is the most challenging component of the research. This theme consists of four subcategories: recruitment and employment of human resources, human resources training (subcategories: lack of knowledge, attitude and clinical skills of employees, academic education, initial training, in-service training), welfare and livelihood issues of employees (two subcategories: inadequate salaries and benefits and work hardship), and human resource motivation (two subcategories: material and spiritual motivation):

“Now you see that people who are working in administrative units such as quality control and other places mostly have relationships to go there.” (Interviewee 30) (lack of meritocracy in selecting staff personnel)

“Unfortunately, the arrangement of personnel is such that someone with low motivation comes with low motivation or someone with low motivation sends a young person next to him and transfers this low motivation to him. The young person who just came wants to work and thinks he is working in a daze, and things like that.” (Interviewee 24) (Inappropriate human resource allocation in the bases)

Based on the participants’ experiences, there is a lack of management stability, multiple management, and not enough time for emergency managers to plan long-term. As a result, they take short-term measures and meet immediate needs. For these reasons, the basic problems of the emergency still persist.

“Another problem, sir, is the multiple management; I do not know the number of positions we had; for example, now I am in Ardabil. I have been in Ardabil for 10 years, almost 11 years; I have experienced seven managers.” (Interviewee 12)

“A lot of the training that is done is like this, in my opinion, they hold a workshop, they hold a class, one comes, one takes a nap, one does not listen, one says to write my name, sign for me, and this is not useful. Such trainings are not useful.” (Interviewee 13)

“The number of standard missions is unknown, job fatigue, and the level of tolerance of individuals for missions are not known. Someone who came on shift and performed 10 CPRs in 24 hours has to leave, and who saw five suicides has to leave. It is not at all visible what effect it has on the technician. On his family, on those around him.” (Interviewee 2)

“Emergency problems can be said to be a financial problem. The financial problem is general throughout the country. About the work we do, the emergency department’s income is very low. If we compare it with other departments, organs, and organizations, I would say that its income is very low, without any pretense.” (Interviewee 3)

### Theme three: Infrastructure

One of the most important factors affecting the provision of appropriate services in the pre-hospital emergency system, in addition to skilled and capable human resources, is the presence of appropriate general and specialized equipment, appropriate rescue vehicles, and an appropriate communication system, which we have referred to in this classification under the title of infrastructure. This theme consists of the categories of transportation system and equipment (challenges of ground relief requiring relief, air relief, challenges of specialized and non-specialized equipment and pharmaceuticals), emergency bases (challenges of relief in conditions of homelessness and shortage and improper distribution of bases) and dispatch centers (challenges of human resources and inefficient communication systems).

“We have problems with logistics, human resources, and processes; that is, we have problems in our three main components. Each has its own stories in their field, and all three need to be improved.” (Interviewee 21)

“Many of the medicines that exist are not used because our children do not like to use them or they do not have the patience or our 1050s, our consulting doctors, do not give them good advice or if they do, they do not have the patience to use them.” (Interviewee 24)

“One of our problems now, especially in our city, is that we practically do not have a dispatch in the real sense. When we leave the base and are notified of a new mission, the phone is connected to the hospital security, and there is no more than one telephone operator there.” (Interviewee 9)

### Theme 4: Public education and information

The appropriate and optimal use of pre-hospital emergency services requires informing the recruits about the duties and responsibilities of this organization. Given that the provision of pre-hospital emergency services in Iran is free, more culture is needed to minimize the possibility of abuse of these services. Based on the participant’s experiences in the field of culture building and information, there are challenges that these problems and challenges are divided into two categories: executive challenges (two subcategories of “inappropriate information from the emergency department” and “limited media activity”) and cultural challenges (two subcategories of insufficient awareness of emergency duties and disruption in providing relief).

“Informing is good. With the awareness that you saw, people should know several things, sir. There are these facilities, and there are not. However, do not abuse these facilities. When you announce that anyone with chest pain should have an EKG, and for some reason, you have determined that an EKG is not needed, and you do not do an EKG. The other party comes and complains. The system also gives the other party the right. This party learns that the emergency department is obliged to take an EKG, so next time, it learns that even if someone says, “My heart hurts” (Interviewee 8)

“Then the discussion of introducing emergency to the people and creating a culture and all this is critical because if you give this training to the staff, my staff will be very professional and know all this, but when they go to the scene, if people are not trained, it makes no sense at all, they will not allow them to operate it, as we are facing now, so it is not just internal training, emergencies have not been able to introduce themselves in public training either.” (Interviewee 18)

“The next problem is the so-called cultural problem. This is perhaps common throughout the country: people’s lack of awareness of the emergency room. The so-called call they make to ask for help from us because they do not know which patients to call and which patients not to call is a blessing.” (Interviewee 3)

### Theme five: Service provision process

The service provided in the pre-hospital emergency room is often provided by an ambulance and a two-person team. To provide this service, the consultant physician stationed in the dispatch unit must issue the necessary authorization. All members of society have the right to access these services and receive the desired services. In this context, the challenges in providing these services are categorized based on the opinions and experiences of the participants into four categories: medical guidance challenges (two subcategories of consulting physicians in need of consultation and factors related to technicians), inadequate coverage of services (four subcategories of uncertainty of non-emergency patients, inadequate specialized services for women, poor service delivery in rural areas, and poor response to accidents and disasters), characteristics of the service provided (three subcategories of delayed service delivery, failure to provide service, and commendable services), and the composition of the service provider team (two subcategories of inappropriate team composition and the challenge of women’s presence).

“One is the technicians’ perspective. The technician’s perspective, for example, is that I should call the patient and explain, saying, “Your patient is like this; what should we do, doctor? This is part of the discussion of the technician’s feelings, self-deprecation (lack of independence), and other things. This is one of the reasons.” (Interviewee 18)

Unfortunately, we never had a proper command system in our EMS systems. For example, when the Azad University incident happened, they wanted to send 50 ambulances at once, so they sent an air ambulance. It could have been collected with an ambulance bus and a few ambulances. They sent all the ambulances at once. The Plasco incident happened, and that was it.” (Interviewee 30)

A participant believed that in some cities, women could be present.

“It was Karaj, but the doctor there was not good. It happens in some cities.” (Interviewee 6).

### Theme 6: Interaction

Since the pre-hospital emergency service is the only specialized institution that responds to all emergencies and diseases outside hospitals and medical centers, and considering the scope of pre-hospital emergency services at the community and national levels, an appropriate response by pre-hospital emergency services requires coordination and interaction within and outside the organization. In this context, the participants’ experiences were divided into two categories: intra-organizational interactions (challenges of inappropriate interaction with dispatch, inappropriate interaction with the university, and inappropriate interaction with the hospital) and extra-organizational interactions.

Some participants referred to improving interactions:

“It is good now, it is better now. We had some contact with them before. Not now, because of the meetings, for example, this winter plan, because the police are the hosts, the Red Crescent, the emergency services, the road administration, and the organizations involved are there. They are familiar with our work.” (Interviewee 12)

“These hospital contacts with nurses are a disaster in themselves. The nurses should be justified that the technician brought this patient from his room, which he did not take, or that he was not a citizen. They speak badly in front of people like this; it makes you feel ashamed as if he were a murderer and went there.” (Interviewee 7)

### Theme 7: The supervising organization

Currently, in Iran, these services are provided by the government and under the supervision of the Ministry of Health and the supervision of the country’s Emergency Organization. All participants believed that the supervising organization should be the Ministry of Health. However, the implementers of these services could be organizations and institutions affiliated with the Ministry of Health or independent of the Ministry of Health. Each of the independent emergency organizations, organizations affiliated with the Deputy Health Office, hospital-based services, municipality-based services, private sector-based services, and the Red Crescent Society were examined. Their strengths and weaknesses were categorized, which are detailed in Appendix 1.

One participant believed that becoming an organization should not mean separating from the university and the academic environment: “To strengthen the position of emergency medicine in terms of authority, credibility, and physical development, becoming an organization is necessary and very good, but it does not mean that now it has become an organization and it should completely separate and isolate itself. I think it would be wrong if becoming an organization leads to separation from an academic source. Emergency medicine students are now studying at the University of Education, and tomorrow, emergency medicine students will leave. For example, in applied science, there will be a total decline in knowledge.” (Interviewee 11).

“It is like, for example, if you leave a child somewhere for someone else to raise and pay for. That network manager is not the main caretaker of the emergency department. It is exactly like if you leave your child for someone else to raise. Now, it is true that, for example, they feed him and take care of him, but maybe he does not see the necessary compassion. . It is like I am responsible for solving the network’s problems, but I do not have control over the human resources and equipment. My equipment is in my own hands, and the networks benefit from this, but the emergency department managers are annoyed.” (Interviewee 26)

Some administrators were against handing over emergency services to the private sector due to legal inconsistencies:

“I disagree with handing over emergency services to the private sector. After all, the emergency is a government agency with no income. The government and the state should completely supervise government agencies. It would be better if it were under the supervision of the university itself, but due to the limitations that exist, we are forced to do so for now.” (Interviewee 13)

### Theme 8: Service delivery model

One of the common concepts in a prehospital emergency is the service delivery model or pattern. Accordingly, there are two general patterns: Anglo-American and Franco-German. Accordingly, some participants considered the country’s model Anglo-American, while others pointed out that the service model was unclear. The responses were expressed as codes and subcategories as strengths and weaknesses. (Appendix 1)

“We are more Anglo-American, meaning Scoop and run, and we are more inclined to take the patient from the scene to the hospital as soon as possible.” (Interviewee 16)

“Look, we are neither that nor that, nor Anglo-American, nor Franco-German, nor do we have doctors or technicians like Anglo-Americans.” (Interviewee 24)

“The current system is based on the Anglo-American model, but given the increasing demand for ambulances, the non-urgent nature of some cases, and the overcrowding and congestion of hospital emergency rooms, a hybrid system would be better. Having a doctor or paramedic board the ambulance and using telemedicine technologies can reduce the burden of unnecessary hospital visits.” (Interviewee 29)

## Discussion and conclusion

After analyzing the study data, 65 subcategories, 27 categories, and 8 themes were extracted from about 800 final codes. These themes were: “Laws and Policies,” Human Resource Management,” Infrastructure,” Public Education and Information,” Service Delivery Process,” Interaction,” Trustee Organization,” and “Service Delivery Model.”

The initial findings of the theme indicate the existence of some laws, protocols, and policies that limit effective service in prehospital emergency care in Iran. Laws that either do not exist or exist but are not implemented or do not have the necessary transparency, and each person interprets it according to their taste. Prehospital emergency care still does not have a specific organizational structure among universities across the country, and legal, financial, and support issues are not very clear, leading to taste-based approaches. This finding is consistent with the results of a study by Irie et al., which was conducted in the prehospital emergency care of Golestan province, where organizational charts and inappropriate administrative rules and regulations were also mentioned as challenges to providing desirable services in the emergency care [6]. In Heidari’s study, the weakness of the laws supporting paramedics was pointed out as a factor for the weakness of paramedics’ assistance (due to unconscious fear of the consequences of performing certain medical procedures) and the interventionist and aggressive actions of the public against paramedics in traffic accidents [13]. Nielsen, who studied the status of prehospital care in 13 low- and middle-income countries, mentioned that one of the important obstacles to the lack of EMS development in these countries was the emergency revenue and budget, the lack of leadership within the system, and the lack of standard laws and regulations, and suggested that the EMS system in these countries requires a legal budget, greater integration and coordination between existing services, and improved management and leadership [14].

Another important finding in this area was the lack of clarity on general programs and policies in prehospital emergency care. Based on the researcher’s findings and experiences, these policies depend on the individual. Each head of the organization has addressed a specific issue in each period, and after the end of his management period, another issue has become prominent in the organization. In these policies and macro-plannings, special attention has not been paid to the issue of prevention and the importance of prehospital services, and this is due to the unknown nature of emergency services among the officials and senior managers of the health system. Appropriate prehospital emergencies are preventive measures against chronic and long-term complications and injuries of injuries and diseases. However, the treatment-oriented policy instead of prevention-oriented has also had its effects on this part of the health system, and the neglect and neglect of prehospital emergency services in the past decades is one of the effects and consequences of these types of policies and plans. In the study by Irie et al., titled “Explanation of the concept and challenges of providing preventive and care services in prehospital emergency: a qualitative study,” conducted in Golestan Province, the lack of a comprehensive and organized program for primary prevention in prehospital emergency care was also noted [6].

The second theme based on the findings was human resource management. Human resources and employees are any organization’s main assets, especially those that provide emergency, vital, and specialized medical services. Services that cannot be entrusted to a machine, robot, or non-professional person due to dynamic, fluid, unbalanced, and unpredictable conditions. Therefore, attracting and employing human resources appropriate to these conditions, training appropriate to these professional services, maintaining and maintaining the knowledge and skills of these people, providing motivation and livelihood for these employees, and ultimately evaluating the performance of these people is one of the fundamental factors in providing effective and timely emergency services. Components that, unfortunately, had many weaknesses and shortcomings in the researcher’s findings about each of them throughout the country. The researcher’s findings included weaknesses and shortcomings in all ranks of employees, including Dispatch, base and ambulance, and administrative headquarters. The most important challenge is the shortage of human resources throughout the country, which was also mentioned in the study by Alinia et al. as a barrier to prehospital services [15]. The study by Jafari, Eiri, Bayrami et al. also mentioned the shortage of human resources in prehospital emergency care [6, 16, 17]. Hosseini also pointed to the weak capabilities of dispatch staff for various reasons as a factor delaying prehospital response [18]. It seems that one of the reasons for this educational deficiency can be seen in the non-expert view of decision-makers and senior educational managers towards the prehospital field and the dominance of the “carrier and tiger” philosophy, which states that this field does not require any special expertise, while based on existing studies in successful and advanced emergency systems in the world, people working in the prehospital field are among the most experienced and capable medical personnel. Now, considering the significant mortality rate of traffic and cardiovascular accidents in the country, shouldn’t we think that by employing capable human resources in prehospital emergencies, many of these preventable deaths can be prevented, and the cost of the health system can be reduced? Guiding and leading an organization to succeed and provide desired services requires competent managers. Managers are from the organization themselves or have high managerial capabilities. Based on the findings, one of the important challenges in this area was the incompetence of senior and middle managers of prehospital emergency centers, which aligns with the findings of Alinia and colleagues. In his study, he referred to empirical management [15]. Medical management is one of the problems in this area. Why shouldn’t a technician and expert in this organization think he can be the head of an emergency center? Do physicians have higher management skills than those with a bachelor’s degree who have worked and gained experience in this system for 20 years? In line with the present study, Arabi also pointed out the concept of physician dominance as one of the extra-professional factors affecting nurses’ participation in health system policy-making [19]. In the study by Hughes, it was also stated that although in most countries, the senior executive management of the Ministry of Health or the leading public health managers are physicians, equal opportunities can be created for the participation of other groups. Unfortunately, organizational systems distort the path of participation by creating unequal levels in the Ministry of Health or allocating unequal resources to each position [20].

The third theme of the research was related to the field of emergency infrastructure. In this area, problems and weaknesses existed in the number, ownership, and physical conditions of emergency bases, ground transportation systems, equipment, and dispatch centers. In the past decades, former managers, for various reasons, especially in metropolitan cities, especially Tehran, would set up an emergency base with a wireless, an ambulance, two technicians, and a building. Alternatively, a room in one of the fire stations or medical centers was allocated to the emergency department with minimal amenities. Therefore, one of the requirements of any emergency system is to have a safe and appropriate location with equipment and to be located at an appropriate point regarding access to roads and the population covered. In this regard, Hosseini, in his study, considered the development of emergency bases and changing indicators as a requirement for reducing time intervals in prehospital emergency care [18]. Bayrami et al.‘s study also noted the existence of non-independent, unsafe, and inappropriate emergency stations in Mashhad [17].

Other issues related to this area mentioned in the findings include the shortage of ambulances and the exhaustion of the ambulance fleet across the country. Pourshikhian’s study also mentioned the shortage of ambulances and stations as a cause of delayed response and a cause of violence against prehospital emergency personnel [21]. In his study, Jafari also mentioned the limited resources and equipment in prehospital emergency services [16]. Airy’s study also mentioned the shortage of equipment, exhaustion of ambulances, and inappropriate arrangement of facilities as challenges to providing desirable prehospital services [6].

Another important issue in the field of infrastructure is communication and telecommunication systems and related software. Despite the development of communication and telecommunication technologies worldwide and in Iran, the prehospital system still lacks some basic facilities common in successful prehospital systems, such as automatic phone identification and caller location detection. There are still shortcomings and disruptions regarding wireless and mobile phone coverage in some parts of the country. In the study by Alinia et al., this issue has also been referred to as the concept of inefficient technology [15].

Other important issues in this area include the challenges related to Dispatch. In some parts of the country, Dispatch does not exist in the real sense, or it is done with minimal workforce and traditional equipment to coordinate and dispatch ambulances. Based on comparative studies in leading countries, emergency dispatches have been combined or used a single emergency number. This leads to coordination and appropriate response at the right time. Therefore, creating a joint dispatch was one of the research findings in the proposed model, which is in line with Heidari’s suggestions [13]. Also, measures should be taken to increase personnel retention in the dispatch unit. Because project personnel were used in this section, these people left the system after completing the project, while they had gained good experiences in this field. In addition, their experience has no use elsewhere.

The fourth theme was the challenges related to public education and information. Because prehospital emergency services in Iran are free, as in some countries, there is a possibility of abuse and unnecessary calls or nuisance calls. Due to the vital nature of prehospital measures and the limited resources in this area, it is essential to use these services appropriately. Insufficient public awareness of emergency services leads to increased calls and, as a result, the loss of real missions. In the study by Alinia et al., public intervention on the scene was mentioned as a deterrent to prehospital services [15]. In the study by Pourshikhian, Bayrami, et al., people’s unfamiliarity with EMS services and the expectation of receiving services outside the job description were also mentioned [17, 21]. In the study by Jafari, intervention by non-professionals was also mentioned, as well as a lack of awareness and culture of appropriate use of EMS services [16].

The fifth theme that emerged from the findings was the service delivery process. One of the important components in the prehospital service delivery process is the medical director or consultant physician. Prehospital literature suggests that the most knowledgeable and committed medical care professionals should be selected as consultant physicians. A physician with experience in a hospital setting who is trained and educated in emergency medicine and trauma, anesthesia, critical care, trauma surgery, or prehospital care is the best candidate for the position of consultant physician. The consultant physician should be involved in the recruitment and training of staff. He or she should also be involved in educational needs assessment, monitoring staff in-service training, developing and revising protocols, striving for quality improvement, reviewing reports, providing direct feedback to the treatment team, and debriefing in critical incidents [22]. However, in the present study, it seems that there is no proper interaction between the consulting physician and the technicians, and the consulting physician has not been able to establish his position as an influential element in the process of providing medical and consulting services in the prehospital emergency department in Iran. In Jafari’s study, this issue has also been referred to as insufficient trust between the physician and the technician [16].

Another important finding related to the service provision process is the existence of people needing ambulance services but not for emergencies, such as people experiencing homelessness and people who cannot take care of themselves. Therefore, it is necessary to respond to the needs of this segment of society by creating insurance mechanisms. In line with the present finding, Ebrahimian also pointed out in his study the existence of such cases as an influential factor in the decision-making of technicians to transfer to the hospital [23]. Also, considering that half of the country’s population is women and there is a need to respond to the specific needs of women by cultural conditions, the presence of a female technician in the ambulance seems essential.

The sixth theme indicates the existence of some inconsistencies and inappropriate interactions within the organization and outside the organization. Considering that prehospital measures require cooperation and coordination of several intra- and extra-departmental units, these inconsistencies affect the appropriate prehospital response. The findings indicated that in some cases, there is no proper communication between technicians and dispatch operators, one of the reasons for which is the demarcation of each base’s operational area and the dispatch operator’s lack of knowledge, leading to these inconveniences. Therefore, it was suggested that dispatch operators should also be present in the field on a rotational basis or that they learn urbanism. Another finding is the inappropriate interaction between technicians and hospitals, which sometimes delay the patient’s admission, which was also mentioned in the studies of Pourshikhian, Jafari, Irie et al. [6, 16, 21]. Therefore, creating a communication platform and increasing interactions between hospital and prehospital officials helps reduce these tensions. In his study, Hosseini considered the existence of coordination between emergency units, such as the consulting physician, the operation control center, the hospital, and other relief units, such as the police and fire brigade, to be effective in reducing the response time of prehospital emergency medical services [18]. Of course, these inconveniences and lack of coordination are decreasing. However, they have not yet reached the desired conditions, and one reason for this is the personality characteristics of individuals, the movements and rotation of personnel in each organization, and the ignorance of individuals in organizations about their duties in the incident. This requires the retraining of new personnel. In this case, we can also mention organizational biases at the top of each organization’s managers, who are unwilling to interact and cooperate.

The seventh theme deals with the challenges of organizations in charge of implementing prehospital emergency medical services. In different countries, numerous organizations and institutions provide these services. However, in Iran, only the National Emergency Organization provides these services throughout the country through the universities of medical sciences. Given that an institution provides these services, That institution depends on the country’s budget, and within that institution, only prehospital services are not valued; the provision of services has faced many challenges; therefore, changing the model and entrusting part of these services to capable organizations, like in other successful countries, seems to be able to reduce some of these challenges. Of course, there is a need for careful monitoring of these transfers; otherwise, the experiences of outsourcing and the lack of monitoring in Iran may increase the challenges of this sector. In the study by Bayrami et al., the lack of independence of the emergency department as an independent organization and the lack of a specific trustee were mentioned with the concept of structural challenges. This study was conducted before the organization of the emergency department [17]. Considering the common language between the hospital and the prehospital, the hospital-based model is suitable for some regions of the country. Because skilled medical personnel are present there, physical facilities, equipment, and the possibility of training in a hospital environment are available. Of course, this plan must be implemented as a pilot first and modified or expanded after evaluation. The second method is for the municipality to provide these services, especially municipalities that are in good financial and logistical conditions. The important point in all methods of delegation and outsourcing is the development of an appropriate delegation law and, more importantly, the supervision of its implementation by the country’s emergency organization.

The last theme of the findings deals with the service delivery model. Considering the strengths and weaknesses of each of the Anglo-American and Franco-American models and that other countries also use the hybrid model, the researcher concludes that, considering the diversity of personnel, geographical conditions, road traffic, hospital conditions in the country, and other reasons, continuing the Anglo-American method and simply providing BLS services no longer meets the needs of patients, personnel, and the country’s conditions. Instead, it is necessary to develop ALS services in addition to BLS services and provide services with a hybrid model. The personnel body is incompatible with the existing model in which they are simply carriers and carriers. He believes that in this model, their literacy and knowledge are unimportant, and their decision-making power is denied. In this regard, Sorani also pointed out in his study that the traditional choice of the scoop-and-run strategy as the dominant approach to prehospital emergency response to disasters, regardless of the level of the incident, has reduced the quality of service provision and reduced prehospital emergency services to merely transporting patients to medical centers [24]. In this regard, Maleki et al.‘s study also noted that Di Bartolomeo et al. concluded that a rescue team consisting of physicians and a helicopter is more effective than a rescue team consisting of medical technicians and a BLS ambulance [25]. Bahadori et al.‘s study also noted that although the results have not confirmed the superiority of one service delivery model, mortality rates are significantly lower in systems where a physician is present on the scene [26].

Since about 20% of prehospital emergency missions in Iran are related to trauma and traffic accidents [27], the need to develop and provide specialized services seems essential. It is also recommended that physicians be used in the field if necessary and that telemedicine systems be strengthened and widespread. Existing forces should be empowered, and, by their capabilities, the necessary authorities should be delegated to them. According to the results of a study by Ebrahimipour et al. in Mashhad, one of the reasons for the transfer of non-emergency patients to the hospital by EMS is the absence of doctors on the scene and in the ambulance and the uncertainty of consulting doctors about the patient’s condition [4]. Regarding the one-stage or two-stage dispatch method, two-stage dispatch reduces the costs of sending advanced teams for non-emergency cases and cases that only require transportation. The opposition group believes that the two-stage system is inherently inefficient, reduces managers’ flexibility to coordinate resources with needs, and that each ambulance typically requires two professionals [28]. The country’s economic and cultural conditions do not allow two-stage dispatch to provide services. Therefore, considering the good experience of motor ambulances in prehospital emergency in Tehran [27] or the single-responder system (special cars, motorcycles, bicycles) in Sweden and other countries [29], it seems that motor ambulances can replace the two-stage dispatch system.

The last point in the discussion is related to evaluating and monitoring individual and organizational performance. The findings indicated no correct method and tool for monitoring and evaluation, whether individual or organizational. In the individual and personnel field, monitoring and control are carried out to catch and punish, and corrective feedback from the performance of the quality control unit is not noticeable in improving the system and has only led to staff dissatisfaction. In the study by Airi et al., the weak system of monitoring and evaluating staff was also mentioned as another challenge in prehospital emergency care [6]. One of the methods of evaluating the capabilities of personnel is creating a ranking plan, which has not yet been adequately implemented in prehospital emergency services throughout the country. The ranking plan aims to create motivation and competition, find talent for key positions, and improve and enhance the quality of prehospital emergency services. The lack of a performance monitoring system and a structure for scientific promotion in prehospital emergency services leads to a lack of need for scientific promotion and sufficient skills among employees, and people’s health is seriously affected. On this basis, while creating a profound change in the quality of prehospital emergency services, employee rankings will also lead to increased satisfaction. This system is not under external supervision in the administrative and organizational fields. When the executor and the supervisor are from the same body or the supervisor does not have the authority and power to deal with, change, and correct the conditions, these evaluations and supervisions only lead to the submission of several pages of reports and changes and corrections of procedures and processes are not made. Of course, in this regard, implementing the prehospital emergency accreditation system is a positive step, but it still takes a long time to achieve the ideal result. In this regard, Sorani also pointed out in his study the weakness or lack of evaluation of organizational performance in the process of distributing and evacuating injured persons in accidents and disasters due to the inadequacy of the process of recording and recording the actions taken and the conditions of the scene and the injured person [24].

This study has certain limitations, including its qualitative nature, which may limit generalizability. Additionally, the sample was restricted to specific stakeholders and may not reflect the views of all EMS users across the country.

### Conclusion

The current service provision model appears insufficient in meeting the evolving needs of society. Therefore, it is recommended to consider enhancing organizational independence at the levels of the Ministry and universities, exploring hybrid service delivery models, and piloting the involvement of other institutions such as hospitals, municipalities, and the private sector in providing prehospital emergency services in selected regions. Evaluating the strengths and weaknesses of these approaches, comparing them with the existing system, and reflecting the results at the national level could contribute to the future development and improvement of the prehospital emergency care system in Iran.

## Supplementary Information

Below is the link to the electronic supplementary material.

Supplementary Material 1

Supplementary Material 2

## Data Availability

The datasets generated and analyzed during the current study are available from the corresponding author upon reasonable request.
