# Supplementary material for: Survey of the situation of the prehospital emergency medical services system in Iran
Source: BMC Emerg Med. 2025 Oct 29;25:219. doi: 10.1186/s12873-025-01350-5 (PMC12573970; doi:10.1186/s12873-025-01350-5)
Supplement: Supplementary file 1 — Supplementary Material 1 [file 12873_2025_1350_MOESM1_ESM.docx]

**Appendices:**

**Appendix 1- Codes and concepts extracted from the qualitative study**

| **Theme** | **Category** | **Subcategory** | **Code** |
| --- | --- | --- | --- |
| **Rules**  **And  Policies** | Policy making | Treatment precedes prevention | Lack of attention to the importance of PHC and emergency  Treatment-centric policy instead of prevention-centric  General laws and policies dependent on the individual  Emergency care is a neglected sector of the health system |
|  |  | Specialization-oriented educational system | Specialized educational system |
|  |  | Centralization | Centralized emergency structure in the country |
|  | Rules and protocols restricting service | Lack of transparency in administrative and financial laws | Lack of transparency of emergency financial resources  Incoherence in the implementation of approvals  Lack of a uniform organizational chart  Unclear supporting laws  Lack of approved instructions  Non-transparent laws and guidelines  Existence of some incorrect indicators |
|  |  | Professional and guild rules | Failure to implement approved laws such as the Law on Promoting Productivity and Efficiency  Non-implementation of the Hard Jobs Act |
|  |  | Lack of SOP | Unclear work standards  Long and unnecessary documentation process  Revision of processes  Lack of SOP |
|  |  | Laws restricting service | The imposition of cumbersome regulations (such as not transferring patients to the private sector, limited ambulance insurance coverage, lack of financial resources, weak laws protecting technicians (driving, accidents, assaults, etc.) |
| **Human Resources Management** | Recruitment and employment of human resources | Non-specialized human resources (technicians) | Shortage of specialized human resources  Limited recruitment  Distortions in the recruitment process  Lack of meritocracy in the selection of staff personnel  Existence of different forms of recruitment  Dual-site workforce  Non-native workforce  Improper arrangement of human resources at the base  Unfair recruitment and distribution of personnel between centers  Presence of non-specialized personnel in ambulances  Decrease in nurses' acceptance of emergency services |
|  |  | Specialist human resources (doctors) | Lack of adequate medical personnel  Unattractiveness of emergency care for physicians  Cost-effectiveness of having a specialist in the ambulance  Ability to perform advanced medical procedures  Relieves responsibility and provides peace of mind for the technician  The presence of a physician leads to better decision-making on the scene  Reduces the burden of unnecessary visits to the emergency department  Reduces the technician's sense of independence |
|  |  | Emergency center managers | Lack of expertise and competence  Multiple management  Non-observance of management hierarchy  Emergency ladder of individual advancement  Medication management  Multi-job emergency managers  Lack of organizational affiliation among managers  Priority of individual interests over organizational ones  Instrumental use of ambulances  Populist thinking instead of cost-effectiveness thinking among managers  Management based on personal orientation  Bus company (outsourcing management) |
|  | Human Resources Training (Educational and Skills Gap) | Lack of knowledge, attitude and skills of personnel | Technician's rest-oriented attitude towards the profession and the base  Insufficient personnel competence  Weakness in general skills of personnel  Weakness in specialized skills |
|  |  | Inadequate academic education | Underqualified teachers  Admission without regard to land use planning  Inefficient teaching methods  Defects in the curriculum of the emergency department  Defects in the student selection process  Inefficient upstream policies and laws  Inadequate infrastructure  Graduates lacking capabilities |
|  |  | Inadequate initial training | Inadequate training of newly hired personnel |
|  |  | Weakness of in-service training | Lack of capable prehospital instructors  Inadequate educational equipment  Lack of motivational factors  Inappropriate interaction with educational centers  Inappropriate educational methods  Non-applied educational content |
|  | Welfare and livelihood | Inadequate salaries and benefits | Injustice in payments  Dissatisfaction with employment type  Non-payment of arrears  Non-financing of personnel  Lack of job benefits  Injustice in distribution of personnel welfare services  Lack of welfare facilities |
|  |  | Difficulty of work | Non-emergency missions  Excessive missions  Job stress  Irregular sleep and eating schedule  Driving stress  Unrecognized psychological effects of the job on personnel  Working in an unpredictable environment  Violence against emergency personnel  Physical injuries  Lack of security at the scene  Traffic problems |
|  | Human resource motivation | Material motivation | Low staff income compared to other organizations  Reduced motivation due to lack of funding  Decreased work motivation due to unfair payments  Falling spiritual motivation due to inadequate income |
|  |  | Spiritual motivation | The hospital does not see the capabilities of the staff  Dismotivation due to disrespect for elders  Inappropriate social status  Lack of importance of knowledge and skills of individuals  Inappropriate incentive and reward system for work  Lack of recognition of the capabilities of the staff in the system  Lack of educational promotion conditions  Unclear career path for staff  Lack of importance of educational degrees in the emergency department  Non-supportive management  Lack of distinction between good and bad |
|  | Evaluation and monitoring | Monitoring individual performance | Incorrect monitoring and control method  Weak monitoring of mission quality  Weak monitoring of technician performance  Wrist grabbing instead of arresting (quality improvement)  Lack of performance evaluation system |
|  |  | Monitoring organizational performance | Lack of external oversight of emergency performance  Lack of accreditation system  Lack of audit system  Weak culture of accountability |
| **Infrastructure** | Transportation system and equipment | Ground relief needs relief | Unprofessional purchase of ambulances  Decentralized maintenance and repair  Ambulance shortage  Ambulance wear and tear  Improper distribution of ambulances  Introduction of new ambulances (strength)  Ambulance engine (strength) |
|  |  | Air relief | Non-specialized air rescue  Proper development of air rescue |
|  |  | Specialized and non-specialized equipment challenges | Non-specialized purchase of equipment  Lack of specialized and non-specialized equipment  Improper distribution of equipment |
|  |  | Medical supplies | Improper process of drug consumption  Lack of medicine  Lack of proper equipment for drug storage |
|  | Emergency bases | Homelessness relief | Non-specialized ownership  Improper safety  Improper physical conditions of the base |
|  |  | Shortage and improper distribution | Inappropriate distribution  Lack of base |
|  | Dispatch Centers | Human resource challenges | Lack of manpower  Insufficient dispatcher skills  High turnover of manpower in the dispatch unit  Weakness of laws supporting dispatchers  Uncertain triage and dispatch protocols |
|  |  | Inefficient communication system | Lack of dispatch center  Problems with the telecommunications network  Lack of balanced quantitative and qualitative development  Interruptions and discontinuities in service provision  Using the traditional system |
| **Challenges of public education and information** | Implementation challenges | Inappropriate information from emergency services | Increasing public expectations of emergency services by emergency officials |
|  |  | Limited media activity | Weakness in creating awareness about emergency services  Weakness in public education |
|  | Socio-cultural challenges | Insufficient awareness of emergency duties | Insufficient public understanding of emergency duties  Dispatcher's lack of tolerance for questions and answers |
|  |  | Interference in timely relief | Interference with appropriate treatment  Unnecessary calls  Service abuse  Telephone harassment |
| **Service delivery process** | Challenges of medical leadership | Consultant Physician Needs Consultation | Shortage of manpower  Doctors on demand  Doctors in love or graduated  Lack of training and experience  Lack of motivation  Distrust  Weak laws  Irresponsibility |
|  |  | Technician Factors | Belief in inefficiency  Resolving legal problems  Formal advice  Doubtful sense of independence  Not providing a suitable communication platform  Recorded tapes  Customized treatments |
|  | Inadequate coverage of services | Uncertainty of non-emergency needs | Homeless patients  Disabled and non-emergency patients |
|  |  | Insufficient specialized services for women | Loss of specialized services for women  Cultural tension |
|  |  | Weak service delivery to rural areas | Inadequate coverage of services in villages  Problems in locating in villages |
|  |  | Weak response to accidents and disasters | Dispatch of personnel and equipment not commensurate with the level of the incident  Lack of emergency preparedness in response to incidents and disasters  Unclear organization in response to disasters  Lack of specialized teams in response to incidents and disasters  Inadequate access to hospitals |
|  | Features of the service provided | Delayed service | Long response time  Delay in response |
|  |  | Failure to provide service | Unprincipled actions  Unregistered medical procedures  Failure to perform duties  Providing unsatisfactory services  Choice-based medical procedures  Failure to comply with professional ethics  Technician's abuse of public awareness |
|  |  | Commendable service | Public satisfaction  Appropriate approach |
|  | Composition of the service provider team | Inappropriate team composition | Insufficient composition of the ambulance team  Inappropriate arrangement of the ambulance team  Inconsistent personnel levels  Necessity of a doctor's presence in the ambulance |
|  |  | The challenge of having a female technician in the ambulance | Limited physical fitness of the female technician  Lack of security for the female technician  Insufficient cultural conditions for the presence of the female technician  Increased mission with the presence of the female technician  Creating expectations from the system for selecting the type of technician |
| **Interaction** | Intra-organizational interaction | Inappropriate interaction with the dispatch unit | Inappropriate interaction between technicians and dispatch operators |
|  |  | Inappropriate interaction with the university | Emergency department neglected in universities  Improper allocation of emergency funds in universities  Problems in the transfer and allocation of funds  Interference in the supervision of private ambulances  Improper interaction with the Deputy Health Minister in the provision of ambulance services |
|  |  | Inappropriate interaction with the hospital | Improving interactions with the hospital  Challenging patient handover and transformation  Hospital emergency department chaos and refusal to accept patients  Inappropriate interaction between hospital emergency physician and technician  Disagreement between dispatch physician and hospital physician |
|  | External interactions | Inappropriate interaction between relief organizations | Inter-organizational interactions dependent on individuals and factions  Delays of relief organizations  Poor interaction with relief organizations  Inefficiency of social emergency services  Inappropriate distribution of equipment and authority between relief organizations  Parallel work in responding to accidents and disasters  Improving interactions with relief organizations on the scene |
| **Trustee organization** | Emergency Organization (independent organization) | Strengths | Improving the status of emergency services in the country  Integrated emergency management throughout the country  Independence and agility of emergency services  Reducing bureaucracy in emergency services and universities  Reducing discretionary actions  Increasing bargaining power at the university level  Financial independence of emergency services  Appropriate distribution of financial resources |
|  |  | Weaknesses | Unsettled organization  Organization as a double-edged sword  Invisible impact of organization  Dualism in organization  Increased problems in case of complete independence at the university level  Lack of necessary infrastructure for organization  Deputy treatment of emergency roadblock  Sabotage and resistance to emergency independence |
|  | Deputy Director of Treatment (Affiliate Organization) | Strengths | Management coherence  Better coordination between different units |
|  |  | Weaknesses | Lack of fluidity  Uniformity of emergency services throughout the country  Dependence of emergency on the country's income conditions  Lobbying in budget allocation  Challenging health network  -Instability in planning and decision-making  -Multiple management at the network and county levels  -The role of the network's stepmother  -Emergency on the way child  -Incoherence in supervision |
|  | Hospital-based | Strengths | Better resource management for both organizations (hospital and emergency)  Improvement of the livelihood of emergency personnel  Providing for the shortage of human resources for both organizations  Saving costs  Using the capacity of hospital personnel in the upcoming crisis Hospital  Improving medical guidance  Improving information flow  Better continuity of services  Increasing and maintaining staff skills  Creating a platform for retrograde training  Improving interactions and understanding mutual conditions |
|  |  | Weaknesses | Lack of central general hospitals in large cities  Challenge of patients outside the hospital's catchment area  Asymmetrical distribution of hospitals across cities  Lack of appropriate infrastructure  Eliminating emergency demands from hospitals  Increasing existing costs  Increasing hospital workload  Different hospital and pre-hospital training  Limited acceptance of 115 by hospital staff |
|  | Municipality | Strengths | More appropriate urban management  More appropriate coordination and response in urban crises  More appropriate response and people's satisfaction  Easier manpower supply  Improvement of the condition of emergency facilities  Reduction of costs  Improvement of personnel's financial and welfare conditions  Finding a specific custodian for emergency services  Integrated urban services |
|  |  | Weaknesses | Different types of services  Lack of common language  Different education and training levels  Different decision-making and action priorities  Impairment in the guidance and supervision of the specialized medical team  Existence of structural and legal problems  Unfair distribution of resources in the country  Increase in existing problems and regression of emergency services  Decrease in service quality  Lack of coherence |
|  | Private sector | Strengths | Reduction in bureaucracy  Easier manpower supply  More authority of the manager over personnel  Reduction of costs  More productivity of corporate personnel |
|  |  | Weaknesses | Weak supervision of private sector performance  Lack of job security  Increase in personnel's financial and welfare problems  Unmotivated personnel  Reduce in service quality  Increase in staff and public dissatisfaction  Maintenance Inadequate equipment and ambulances  Inefficient recruitment of personnel  Relationships in outsourcing services to the private sector  Differences in outsourcing laws  Contrary to the Government Services Law  Inability of Iranian companies to provide full service |
|  | Rescue and Relief Organization (Red Crescent Society) | Strengths | With extraordinary capacity  Interaction with the fire department or Hilal Yes, integration No |
|  |  | Weaknesses | Unclear organizational structure  No obligation to be accountable  Non-specialized organization  Reduction in service quality  Existence of a competitive environment between the two organizations |
| **Service Delivery Model** | Anglo-American Service Model | Strengths | Compatibility of existing equipment and manpower with this model  Compatibility of people's culture with this model  No preference between the two models over each other |
|  |  | Weaknesses | Unwillingness of personnel to perform basic measures  Unresponsiveness of the system |
|  | Franco-German Service Model | Strengths | Managers' tendency towards providing specialized services  People's acceptance of receiving specialized services  Reduction of the burden of hospital visits |
|  |  | Weaknesses | Inability to provide financial resources  Inability to provide specialized equipment for doctors  Inadequate training of doctors in accordance with emergency needs  Shortage of specialists and inability to employ them in the field  Increase in calls with the presence of a doctor in an ambulance |
|  | Hierarchical dispatch system | Strengths | Comparable to resources |
|  |  | Weaknesses | Resource limitations  Waste of time  Increase in costs  Cultural problem |
