# Supplementary material for: Survey of the situation of the prehospital emergency medical services system in Iran
Source: BMC Emerg Med. 2025 Oct 29;25:219. doi: 10.1186/s12873-025-01350-5 (PMC12573970; doi:10.1186/s12873-025-01350-5)
Supplement: Supplementary file 2 — Supplementary Material 2 [file 12873_2025_1350_MOESM2_ESM.docx]

This semi-structured interview guide was designed based on a review of related studies and consultation with specialists and experts in the field.

1. Interview Questions for Executive Managers:

- Can you describe your experience managing prehospital emergency medical services?
- What are the main challenges you face in managing these services?
- What solutions do you suggest for improving these services?

1. Interview Questions for Senior Managers, Policymakers, and Scholars:

- In your opinion, what are the most significant issues facing prehospital emergency medical services?
- How can these issues be resolved?
- What experience or knowledge do you have that can be helpful in this field?

1. Interview Questions for Operational Staff:

- Can you describe your experience providing prehospital emergency medical services?
- What challenges do you encounter at the scene?
- What suggestions do you have for improving services?

1. Interview Questions for Service Users (General Public):

- How was your experience receiving prehospital emergency medical services?
- What problems did you experience while receiving these services?
- What are your expectations from prehospital emergency medical services?
